# Generation of transgenic marmosets expressing genetically encoded calcium indicators

**DOI:** 10.1038/srep34931

**Published:** 2016-10-11

**Authors:** Jung Eun Park, Xian Feng Zhang, Sang-Ho Choi, Junko Okahara, Erika Sasaki, Afonso C. Silva

**Affiliations:** 1 Cerebral Microcirculation Section, Laboratory of Functional and Molecular Imaging, National Institute of Neurological Disorders and Stroke, National Institutes of Health, Bethesda, MD, USA; 2 Department of Applied Developmental Biology, Central Institute for Experimental Animals, Tonomachi, Kawasaki, Kanagawa 210-0821, Japan; 3 Keio advanced Research Center, Keio University, Shinanomachi, Shinjuku-ku, Tokyo 160-8582, Japan

## Abstract

Chronic monitoring of neuronal activity in the living brain with optical imaging techniques became feasible owing to the continued development of genetically encoded calcium indicators (GECIs). Here we report for the first time the successful generation of transgenic marmosets (Callithrix jacchus), an important nonhuman primate model in neurophysiological research, which were engineered to express the green fluorescent protein (GFP)-based family of GECIs, GCaMP, under control of either the CMV or the hSyn promoter. High titer lentiviral vectors were produced, and injected into embryos collected from donor females. The infected embryos were then transferred to recipient females. Eight transgenic animals were born and shown to have stable and functional GCaMP expression in several different tissues. Germline transmission of the transgene was confirmed in embryos generated from two of the founder transgenic marmosets that reached sexual maturity. These embryos were implanted into six recipient females, three of which became pregnant and are in advanced stages of gestation. We believe these transgenic marmosets will be invaluable non-human primate models in neuroscience, allowing chronic *in vivo* monitoring of neural activity with functional confocal and multi-photon optical microscopy imaging of intracellular calcium dynamics.

Optical monitoring of neuronal populations tagged with fluorescent calcium-sensitive molecules has become an attractive way to study brain function *in vivo*, particularly after the development of genetically encoded calcium indicators (GECIs)^1^,^2^. GECI molecules sense calcium influx into excitable cells. Upon calcium binding, GECI molecules fluoresce, constituting a visible marker of cellular function and activity. The most optimized family of GECIs to allow monitoring of neural activity *in vivo* are GCaMP, which are based on a fusion of the calcium-binding protein calmodulin with the green fluorescent protein (GFP)^3^,^4^. Most notably, the new generation of GCaMPs has demonstrated higher sensitivity to individual action potentials in neuronal somata and in dendrites with good signal to noise ratio^4^,^5^. GCaMPs can be directly delivered to discrete areas of the brain via stereotaxic injections of recombinant adeno-associated viruses (AAV) or lentiviral vectors^6^,^7^. These viruses are able to infect non-dividing neuronal cells with apparent low toxicity, and induce stable long-term transgene expression^8^,^9^. However, these methods are invasive, requiring surgery on each animal. In addition, the viruses produce inhomogeneous expression patterns across the infection site, and lead to undesirable expression levels, causing aberrant cell death^7^. Thus, virus-mediated gene transfer can only be used for experiments within a limited time window, and only after careful examination of the relationships between viral tropism, promoter constructs, virus titer, injection volume and preferential gene expression in neurons^8^,^10^,^11^. As an alternative approach, several GECI expressing transgenic mouse lines have been developed^6^,^12^,^13^,^14^,^15^,^16^ to allow the investigation of brain-wide neuronal activity in the living brain under physiological conditions. The derivation of transgenic animal models is presumably a better approach to study physiological processes, as levels of expression of the transgene are endogenously regulated in a way to avoid toxicity effects associated with the local overexpression of virus-mediated transgenes^6^,^16^.

Although it is possible to study various neurophysiological processes with well-established rodent models, the close similarities to humans in terms of genetic and physiological features are undeniable reasons that nonhuman primates are ideal animal models for studying the complex human brain^17^,^18^. Additionally, the recent feasibility of genome modification in nonhuman primates has significantly raised their role and importance in biomedical research^19^. The common marmoset (*Callithrix jacchus)* is a New World monkey that is a clear example of a nonhuman primate with ever growing interest as a model for neuroscience research, in great part due to the successful generation of transgenic marmosets with germline transmission of the transgene^20^.

In the present work, we describe the generation of transgenic marmosets using a lentiviral vector containing GECI molecules of the GCaMP family to infect marmoset embryos. Eight transgenic marmosets expressing GCaMP5g or GCaMP6s were successfully born. Germline transmission of the transgene was confirmed in oocytes collected from two of the founder transgenic marmosets that reached sexual maturity. These transgenic marmosets hold the promise to be a powerful tool for imaging neural activity *in vivo* that constitutes a closer and more accurate model in translational studies of aimed at understanding human brain function.

## Results

### Production of transgenic marmosets

**Figure 1:**
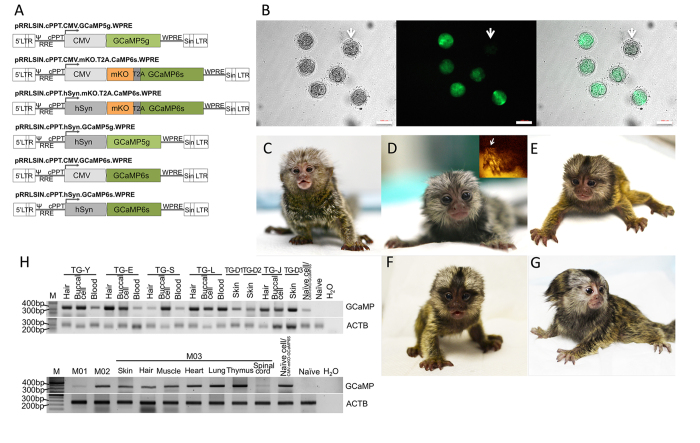
Generation of GCaMP-expressing transgenic marmosets derived by infection of wild-type zygotes with lentiviral vectors. (**A**) Schematic diagram of the lentiviral vectors used. Only the relevant portions of the plasmid are shown. (**B**) Verification of transgene expression in IVF embryos 4 days after lentiviral injection. Left, bright field image. Middle, green fluorescence image shows clear expression of GCaMP. Right, merged image. The arrow shows a naïve embryo. Scale bar = 100 μm. (**C**–**G**) Photographs of the infant transgenic marmosets. Shown are: (**C**) TG-Y (CMV-GCaMP5g); (**D**) TG-E (CMV-mKO-GCaMP6s). The inset shows an epifluorescence image of the left front paw of TG-E (left, arrow) placed against that of a wild-type animal. Note mosaic expression of the inert orange tag mKO in the fur of the digits; (**E**) TG-S (hSyn-mKO-GCaMP6s); (**F**) TG-L (hSyn-mKO-GCaMP6s); and (**G**) TG-J (CMV-GCaMP6s). (**H**) PCR products of the transgene in animals born alive (TG-Y, E, S, L, D1, D2, J, and D3) and aborted fetuses (M01-03) showing the presence of the GCaMP transgene in different tissues. M: DNA marker; Naïve cell/CMV-mKO-GCaMP6s: genomic DNAs extracted from wild type marmoset fibroblasts infected with CMV-mKO-GCaMP6s lentiviruses for positive control; Naïve: genomic DNAs extracted from wild type marmoset fibroblasts for negative control.

To generate GCaMP transgenic marmosets, we utilized high titer lentiviruses that expressed either GCaMP5g or GCaMP6s under the control of two different promoters: the human cytomegalovirus (CMV) enhancer/promoter^21^ for ubiquitous expression, or the human synapsin I (hSyn) promoter^22^ for neuron-specific expression. For GCaMP6s constructs, we added an inert monomeric kusabira-orange (mKO) fluorescent tag for better detection of transgene expression. The lentiviral vectors were named CMV-GCaMP5g, CMV-mKO-GCaMP6s, hSyn-mKO-GCaMP6s, hSyn-GCaMP5g, CMV-GCaMP6s and hSyn-GCaMP6s, respectively (Fig. 1).

Marmoset embryos were collected from donor females either via nonsurgical uterine flushing of naturally fertilized (NAT) embryos, or alternatively via laparotomic follicular aspiration of unfertilized oocytes followed by *in vitro* maturation and fertilization (IVF)^20^,^23^. We performed 52 uterine flushing procedures and successfully collected 108 NAT embryos in 43 of them (82.7% successful flushing, 2.5 embryos/successful flushing). In addition, we collected 787 oocytes from 17 surgical laparotomies (average 46 oocytes/surgery). Of these, 625 (79.4%) successfully matured to the MII stage and, out of this cohort, 551 (88.2%) were successfully fertilized (Table 1).

Following embryo production and collection, 476 IVF and 100 NAT embryos were injected at their earliest possible embryonic stage with high titer lentiviral vectors (6 × 10^8^ − 1.5 × 10^11^ transducing units per ml). NAT embryos were transferred into recipient females on the same day, while IVF embryos were maintained in culture and examined for transgene expression 3–4 days after lentiviral infection. Among the injected IVF embryos, 385 (80.9%) developed successfully *in vitro* and, of those, 345 (89.6%) displayed fluorescence, indicating early expression of the transgene.

To generate transgenic marmosets, 187 IVF and 88 NAT embryos were implanted into 51 and 31 recipient females, respectively. Twenty-two of the recipient females (26.8%, 22 out of 82; eight CMV-GCaMP5g, two CMV-mKO-GCaMP6s, six hSyn-mKO-GCaMP6s, two hSyn-GCaMP5g, three CMV-GCaMP6s, and one hSyn-GCaMP6s) became pregnant and 8 live newborns (one pair of twins, 6 singletons) were delivered naturally at full term (Table 2). Among these 8 newborns, 3 died of unknown causes. One infant (TG-D2) survived for 15 days after birth, while the other two (TG-D1 and D3) died within 24 hours after birth. The remaining 5 newborns, 3 males (TG-S, L and J) and 2 females (TG-Y and E) developed normally. At the present time, the two females reached sexual maturity, while the 3 males are still juveniles.

### GCaMP transgene integration and expression

Transgene integration was confirmed in all 8 transgenic marmosets by PCR using genomic DNA extracted from tissues that could be acquired noninvasively (hair roots and mouth swabs from infants; peripheral blood cells from juveniles, Fig. 1). Transgene integration was further verified in different organs of three miscarried fetuses, including skin, muscle, heart, lung, thymus (Fig. 1). The genomic integration loci of the transgene in the transgenic marmosets were determined by a restriction enzyme-PCR based technique. Multiple copies of integration in the genomic DNA of peripheral blood (TG-Y, E, S, L, and J) and tissue (TG-D1, D2, and D3) were found (Table S1).

**Figure 2:**
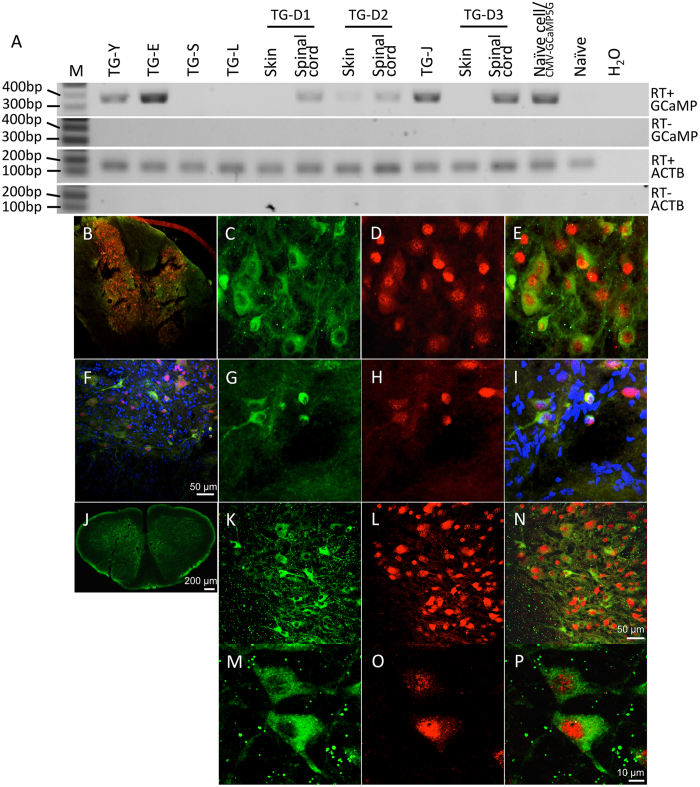
Expression patterns of GCaMP in transgenic marmosets. (**A**) RT-PCR analysis from hair roots (TG-Y, E, L, S, and J), skin and spinal cord tissues (TG-D1, D2, and D3). M: DNA marker; Naïve/CMV-GCaMP5g: wild type marmoset fibroblasts infected with CMV-GCaMP5g; Naïve: wild type marmoset. (**B**–**P**) Cross-section samples of spinal cord tissue from TG-D1 (**B**–**E**), M03 (**F**–**I**), and TG-D3 (**J**–**P**) were immunostained against GCaMP and NeuN (red) or DAPI (blue), respectively. GCaMP is highly expressed in the neurons of gray matter in the spinal cord.

GCaMP mRNA expression levels in hair roots of transgenic marmosets injected with the CMV promoter constructs (TG-Y, D2, E, and J) were confirmed by RT-PCR, but not in animals injected with the hSyn promoter (TG-S, L, D1, and D3), as expected (Fig. 2). Expression of GCaMP mRNA was also determined in spinal cord tissues obtained post-mortem from TG-D1, D2 and D3. As shown in Fig. 2, GCaMP expression was only detected in the spinal cord but not in other tissues, indicating neuron-specific expression of GCaMP under control of the hSyn promoter. Consistently with RT-PCR results, flow cytometric analysis of peripheral blood samples showed GCaMP positive cells in transgenic marmosets injected with the CMV promoter (Fig. S1). Among peripheral blood cells, most of the GCaMP positive cells were granulocytes with a proportion of 10.16% in TG-Y and 14.49% in TG-E, respectively. Transgene expression in the body of the animals with the mKO tag under control of the CMV promoter was confirmed by direct visualization of fluorescence under UV light (Fig. 1). In TG-E, a patchy orange fluorescence was observed throughout the animal’s fur, indicating that not all blastomeres of the NAT embryo were infected with the viral construct. We also examined GCaMP expression by immunohistochemistry in spinal cord tissue obtained from transgenic animals with the hSyn promoter (TG-D1, D2 and M03), and confirmed GCaMP expression (Fig. 2). These tissue samples were also co-stained with an antibody against the neuronal marker NeuN, demonstrating the localization of GCaMP in neurons (Fig. 2).

### Functionality of the GCaMP molecules in transgenic marmosets

**Figure 3:**
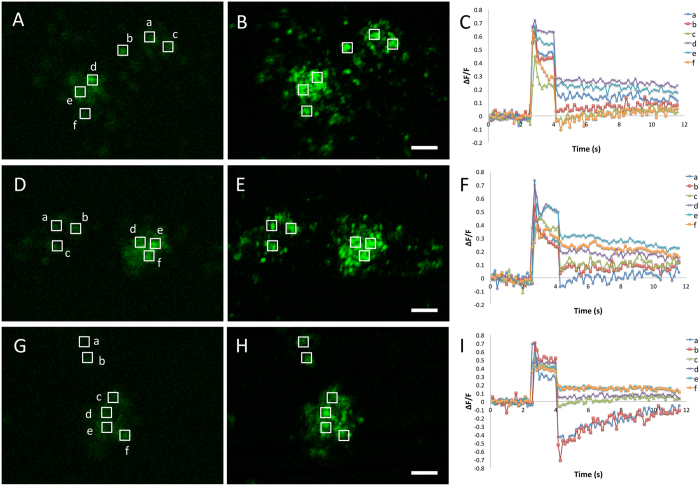
*In vitro* demonstration of the functionality of the transgene in cells derived from a CMV-GCaMP5g transgenic marmoset (TG-Y). (**A**,**B**) Fluorescent images of the cells derived from TG-Y before (**A**,**D**,**G**) and after (**B**,**E**,**H**) stimulation of the cell culture with 1mM ATP added to the medium show robust changes in fluorescence with the ATP-induced increase in intracellular calcium levels. (**C**,**F**,**I**) Time courses of the changes in fluorescence in six example areas (a–f) demarcated in A, D and G. Scale bar = 50 μm.

To test the functionality of the integrated GCaMP protein, primary cells were derived from transgenic marmosets generated using the CMV promoter. We initially derived fibroblasts from skin biopsies obtained from TG-Y and E. Some of the cells exhibited low basal fluorescence under normal culture conditions and a large increase in fluorescence after stimulation with highly-concentrated calcium chloride added to the culture medium (Fig. S2). Likewise, primary cells derived from TG-D3’s spinal cord and maintained in culture exhibited patchy low basal fluorescence before being stimulated with calcium-chloride, and higher fluorescence signal upon stimulation (Fig. S2). Next, a mixed population of cells derived from aspirated follicular fluid collected during oocyte harvesting procedures performed in TG-Y exhibited low basal fluorescence under quiescent conditions (Fig. 3). Stimulation of the cells with ATP added to the culture medium (final concentration of 1 mM) caused a strong increase in fluorescence, indicating functionality of the GCaMP molecules (Fig. 3).

### Germline transmission of the transgene

As two of the founder marmosets (TG-Y and E) reached pubertal age, serum progesterone levels were monitored to detect onset of sexual maturity, established when the progesterone level rose above 10ng/ml^24^. Upon confirmation of an active reproductive cycle, we administered a superovulation-inducing treatment and collected a total of 168 oocytes via surgical laparotomies (118 from TG-Y, 50 from TG-E). Of these, 131 (78%) matured to the MII stage and, out of this cohort, 113 (86.2%) were successfully fertilized by IVF (F1 embryos).

**Figure 4:**
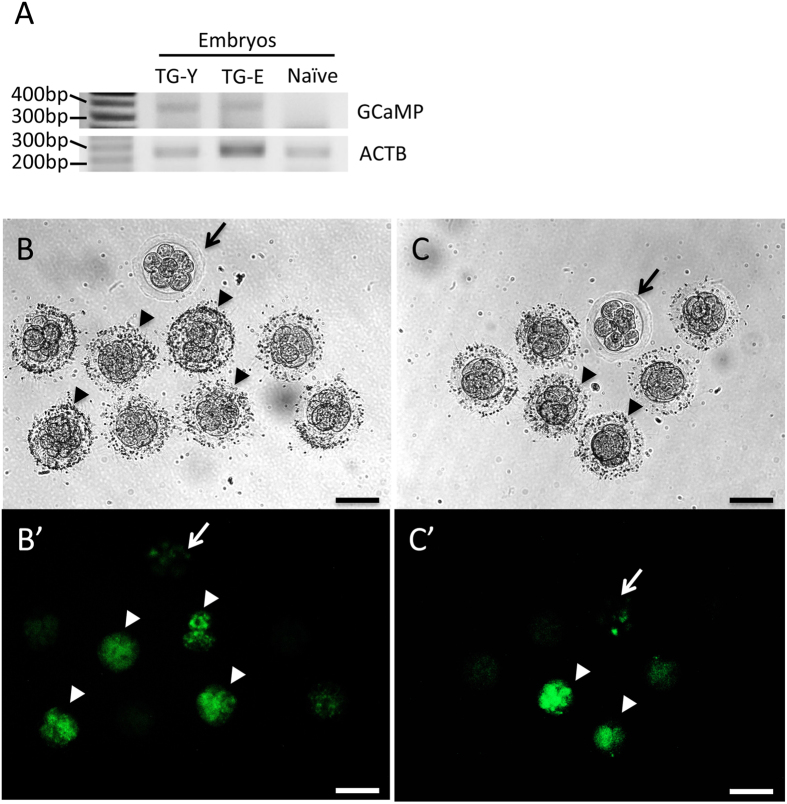
Germline transmission of GCaMP transgene to F1 embryos. (**A**) PCR analysis showing the presence of the GCaMP transgene in F1 embryos generated from TG-Y and E. (**B**,**C**) Bright-field and epifluorescence (**B**′,**C**′) images of F1 embryos. GCaMP positive IVF embryos produced from TG-Y (**B**,**B**′) and TG-E (**C**,**C**′) are indicated by arrowheads. Arrow: naïve embryo. Scale bar = 100 μm.

Among the F1 IVF embryos, 83 (73.5%) developed successfully *in vitro* and of those, 31 (37.3%) displayed fluorescence, indicating early expression of the transgene and successful germline transmission to the next generation (Fig. 4). Twenty embryos from TG-Y and six from TG-E were implanted into 4 and 2 recipient females, respectively. Three of the recipient females (50%, 3 out of 6; one CMV-GCaMP5g, two CMV-mKO-GCaMP6s) became pregnant and are still in advanced stages of their gestation period.

## Discussion

Five different GCaMP-expressing transgenic marmoset lines were successfully generated by infection of naïve embryos with lentiviral vectors encoding CMV-GCaMP5g, CMV-mKO-GCaMP6s, hSyn-mKO-GCaMP6s, hSyn-GCaMP5g and CMV-GCaMP6s. We verified integration and expression of GCaMP molecules in different tissue samples obtained from all eight live births, and from 3 other stillborn infants. Functionality of the transgene was assessed by measuring fluorescence changes in response to a calcium challenge in cultured fibroblasts derived from transgenic marmosets generated using the CMV promoter and in spinal-cord tissue obtained from a transgenic marmoset generated using the hSyn promoter. These results show that transgenic marmosets expressing GECI molecules can be successfully generated, and substantiate the promise of these non-human primates as an invaluable animal model in neuroscience, particularly in experiments aimed at monitoring neural activity and intracellular calcium dynamics with functional confocal and multi-photon optical microscopy.

There are several advantages to using marmosets for creating non-human primate transgenic lines. As demonstrated by Sasaki *et al*.^20^ and also here, germline transmission in marmosets occur two to three times faster than in macaques, due to their short time (15–18 months) to reach sexual maturity. In addition, marmosets are prolific breeders well adapted to living in captivity. Once formed, mature breeding pairs give birth twice a year, as the gestation time is short (143–145 days) and the females can get pregnant again shortly after giving birth^25^. Thus the intergeneration time in marmosets is close to 2 years, allowing for a relatively fast establishment of homozygous transgenic lines free of mosaic expression of the transgene.

Specifically to neuroscience applications, marmosets retain the typical anatomical and functional organization of the primate brain^26^,^27^,^28^, and display many social, cognitive and behavioral functions typical of primates^29^,^30^,^31^,^32^,^33^. Marmoset newborns are developmentally immature, allowing for studies of primate brain development^34^,^35^,^36^ and yet they have the shortest lifespan amongst the anthropoid primates, thus allowing studies of neurodegenerative disorders^37^ to happen within a reasonable timeframe. Furthermore, recent advances in genome editing techniques, such as zinc finger nucleases (ZFNs), transcription activator like effector nucleases (TALENs), and clustered regularly interspace short palindromic repeat (CRISPR)/CRISPR-associated protein 9 (CRISPR/cas9), have facilitated the generation of gene targeted non-human primates^38^,^39^,^40^. More recently, successful generation of IL2RG knockout marmosets with immunodeficient phenotypes have been demonstrated and provided feasibility of multiple preclinical and translational applications^41^. Therefore, marmosets hold much promise in furthering our understanding of the primate brain, and the development of transgenic marmosets expressing GECI molecules in the brain is likely to provide a significant experimental advantage in allowing simultaneous monitoring of neural activity from thousands of neurons *in vivo* using optical imaging technology^15^,^42^.

We used lentiviral vectors to transfer the GCaMP gene into marmoset embryos derived either from natural mating (NAT) or *in vitro* fertilization (IVF). Lentivirus-mediated gene transfer has been recognized as an efficient method to generate transgenic animals, including nonhuman primates^9^,^20^,^43^. We were able to achieve an embryo cleavage rate of 80.9% and a transgene expression rate of 89.6% after subzonal injection of the lentiviruses, indicating the efficient integration of these proviruses into the marmoset genome with minimal interference on embryonic viability. In addition, recent progresses made on the adaptation of assisted reproductive techniques for marmoset monkeys, including controlled ovarian stimulation protocols, *in vitro* maturation and fertilization of oocytes, non-invasive sperm collection, embryo collection from donor females and transfer into recipient females, have strongly facilitated the production of transgenic marmosets and enhanced their value as experimental animal models^23^,^44^,^45^,^46^,^47^,^48^,^49^. By implementing these techniques, we were able to repeatedly recover pre-implantation stage marmoset NAT embryos noninvasively without any residual impact to the donor female’s reproductive health. As well, the use of superovulation techniques enabled us to reliably retrieve a large number of viable oocytes. Compared to NAT embryos, IVF embryos have the main advantage of allowing the virus infection at the single cell stage, which greatly minimizes mosaic expression of the transgene. In addition, the successful use of *in vitro* maturation and fertilization techniques enabled us to reduce the number of animals needed for generating transgenic marmosets significantly.

Validation of transgene inheritance is a necessary step both in the creation of transgenic lines and for rapid expansion of transgenic colonies^50^. Transgenic rodents generated by lentivirus-based gene delivery have demonstrated successful germline transmission and showed similar levels of transgene expression in the progenies, indicating that the provirus is not inactivated during the round of gametogenesis and during development^51^. In line with the rodent study, germline transmission, along with stable expression levels of the transgene in the offspring, was demonstrated in transgenic common marmosets produced by injecting a lentiviral vector containing the GFP gene^20^. More recently, germline transmission was confirmed in a rhesus transgenic model of Huntington’s disease generated by lentiviral transgenesis both in embryonic stem cells generated from the founders, as well as in their descendants^9^,^52^,^53^. In the present study, the two founder transgenic females became sexually mature, and we were able to confirm transgene expression in IVF embryos fertilized with sperm from wild type males. This result shows successful transgene transmission to the next generation. The IVF embryos were transferred to recipient females, which are currently in advanced stages of pregnancy. Therefore, we expect to establish GCaMP expressing transgenic marmoset lines within a reasonably short time frame. Notably, we have determined the chromosomal location of the transgene integration in our founders using a methodology developed recently^54^,^55^. With the now readily accessible genome sequence, mapping studies of retroviral integration sites in cells have uncovered that HIV and HIV-based lentivectors strongly prefer integrating inside actively transcribed genes^56^,^57^. For example, when hematopoietic stem cells of rhesus monkeys were transplanted with MLV or SIV transduced CD43 + cells, a large number of genes were identified with two or more integration events and these genes were deemed common integration sites. A long-term follow up of a large cohort of nonhuman primates has shown completely normal hematopoiesis and lack of any progression towards neoplasia^58^,^59^, raising the possibility that stable transgene expression can be obtained with a relatively low risk of insertional mutagenesis. However, integration site patterns may be cell type dependent, as gene activity impacts integration site selection^60^. Currently, the distribution of lentiviral integration sites in early marmoset embryos has not been fully evaluated. In the future, we will monitor the segregation of transgenes and their expression patterns in the progenies, as we believe such work should facilitate characterization of possible positional effects on phenotype. Additionally, design of safer vectors, including the use of insulating elements to decrease the risk of activation of adjacent genes^61^,^62^,^63^ or novel vectors with different integration patterns^64^, should allow continued progress towards a safer and more effective transgenic technology.

The GCaMP expressing transgenic marmosets were generated under the control of both the CMV and the hSyn promoters. GECI molecules, including GCaMPs, have been used more largely in the field of neuroscience as an effective monitoring system for neural activity-dependent calcium signaling^5^,^6^,^15^. Since calcium signaling is involved in a variety of intracellular signaling pathways, GCaMP is also being applied in human pluripotent stem cells and in transgenic rodents engineered to express GCaMP predominantly in smooth muscle cells, cardiomyocytes, and kidney proximal tubules, thus allowing calcium imaging without additional manipulation^65^,^66^,^67^,^68^,^69^,^70^. Owing to the limited access of tissues from the founder animals, a more thorough characterization of GCaMP expression, as well as a deeper assessment of the functionality of the transgene *in vivo* still remains to be performed. We expect that this work will open up new possibilities for physiologic investigation of calcium dynamics in nonhuman primate models.

In summary, we successfully generated transgenic marmosets that stably express GCaMP under the control of the CMV or the hSyn promoters using lentiviral transgenesis. Germline transmission of the transgene was confirmed in oocytes collected from two of the founder females. These transgenic marmosets may become a useful and practical primate model available for the study of calcium dynamics, particularly for functional optical imaging of neural activity *in vivo*.

## Methods

All procedures were approved by the Animal Care and Use Committee of the National Institute of Neurological Disorders and Stroke (NINDS) and performed in accordance with institutional guidelines.

### DNA constructs and lentiviral production

GCaMP5g and GCaMP6s constructs have been described^4^,^5^. For the CMV-GCaMP5g and CMV-GCaMP6s construct, the open reading frame of CMV-GCaMP5g (pCMV-GCaMP5g was a gift from Douglas Kim and Loren Logger, plasmid 31788, Addgene, Cambridge, MA, USA)^4^ or CMV-GCaMP6s (pGP-CMV-GCaMP6s was a gift from Douglas Kim, plasmid 40753, Addgene)^5^ used to replace the PGK-EGFP sequence in the parental pRRLsin.cPPT.PGK-EGFP.WPRE lentiviral vector plasmid (plasmid 12252, Addgene). For the CMV-mKO-GCaMP6s construct, the humanized monomeric Kusabira-Orange 2 cDNA sequence was obtained from phmKO2-MC1 (mKO, AM-V0145; MBL, Tokyo, Japan), linked via T2A to the open reading frame of GCaMP6s (plasmid 40753, Addgene) and then used to replace the EGFP sequence in the lentiviral backbone vector. For the hSyn-mKO-GCaMP6s construct, the human synapsin I promoter sequence obtained from pDRIVE-hSynapsin (Invivogen, San Diego, CA, USA) was attached to the mKO-T2A-GCaMP6s sequence and subcloned into the lentiviral backbone vector. For the hSyn-GCaMP5g and hSyn-GCaMP6s construct, the open reading frame of GCaMP5g or GCaMP6s was replaced the mKO-GCaMP6s in the hSyn-mKO-GCaMP6s lentiviral vector plasmid.

Lentiviruses were produced through transient transfection of 293T cells with CMV-GCaMP5g or CMV-mKO-GCaMP6s plasmid, along with gag-pol, rev-tat, and VSV-G packaging plasmids^71^. Viruses were harvested daily for 4 days, filtered (0.22 μm; EMD Millipore, Darmstadt, Germany) and concentrated by ultracentrifugation at 18000g for 4 hours at 4 °C, then reconstituted in media. For hSyn-mKO-GCaMP6s, hSyn-GCaMP5g, CMV-GCaMP6s and hSyn-GCaMP6s, ready to use high-titer purified lentiviral vectors were produced by the University of Pennsylvania School of Medicine Vector Core.

### Marmoset embryo collection and lentiviral injection

Adult common marmosets (C. jacchus, n = 46) were selected from the NINDS marmoset colony to be used in this study. The NINDS colony was established in 1997 and it has been maintained in a self-sustaining outbred status by careful selection of breeding pairs that are not used for any other experimental purposes. Donor females (n = 6) were individually and permanently paired with intact males for natural (NAT) embryo collection. Another set of donor females (n = 4) and all recipient females (n = 16) were individually and permanently paired with vasectomized males. The animals are housed in pairs in dedicated cages and maintained on a twelve-hour light/dark cycle. Their diet consists of ad libitum Zupreem canned marmoset food, Purina 5040 biscuits, unfiltered water, and P.R.A.N.G. rehydrator. In addition, the animals are fed daily with various fruit and vegetable treats.

Marmoset embryos were collected from the donor females via nonsurgical uterine flushing (NAT embryos) or via follicular aspiration of the ovaries followed by *in vitro* maturation and fertilization (IVF embryos), as previously described^20^,^23^,^46^,^72^. Prior to the nonsurgical collection of NAT embryos, blood samples (0.1 ml) were taken from the femoral vein of the donor females 1 and 10 days after the injection of the cloprostenol (Estrumate; Schering-Plough Animal Health, Union, NJ, USA), and assayed for serum progesterone concentration using a Progesterone EIA kit (Cayman chemical, Ann Arbor, MI, USA). The ovulation day (Day 0) was determined as the day in which serum progesterone levels rose above 10 ng/ml^24^. Uterine flushing was performed on Days 4–7 after ovulation and the collected embryos were cultured in BlastAssist medium (Origio MediCult Media, Måløv, Denmark) at 38 °C in an incubator in a humidified atmosphere of 5% CO_2_, 5% O_2_ and 90% N_2_. For collection of germinal vesicle-stage (GV) oocytes, female marmosets were subjected to a superovulation treatment and oocyte-cumulus complexes (COCs) were surgically aspirated as described previously^23^. *In vitro* oocyte maturation was performed by incubation in porcine oocyte medium (POM; Cosmo bio Co. LTD, Carlsbad, CA, USA) supplemented with 5% FBS (Invitrogen, Carlsbad, CA, USA), 5 IU/ml FSH (Gonal-f, EMD Serono, Rockland, MA, USA) and 5 IU/ml hCG (Sigma, St. Louis, MO, USA) under the mineral oil at 38 °C in a humidified 5% CO_2_, 5% O_2_ and 90% N_2_. Freshly ejaculated semen was collected in Tyrode’s albumin lactate pyruvate (TALP) medium (Caisson labs, North Logan, UT, USA), washed twice and placed in a incubator for 45 min in a test tube inclined at a 30° angle to allow the sperm to swim up. *In vitro* matured oocytes were inseminated with a final concentration of 5 × 10^6^ sperm/ml for 16–20 h and fertilized embryos were cultured in ISM1 medium (Origio MediCult Meia)^20^,^73^.

One cell to morula stage embryos were placed in 0.25M sucrose supplemented M2 medium (Sigma), and the concentrated lentivirus was injected into the perivitelline space using a FemtoJet 4i and micromanipulator (Eppendorf North America, Inc., Hauppauge, NY, USA).

### Embryo transfer and pregnancy diagnosis

The estrus cycle of donor and recipient animals were synchronized and microinjected embryos were nonsurgically transferred as previously described^23^,^46^,^72^. Pregnancies were detected with an ultrasound scanner 1–30 days after embryo transfer and monitored ultrasonographically monthly after initial confirmation.

### Genomic DNA analysis

Genomic DNA was extracted from tissue samples from all the transgenic marmosets and subject to PCR for transgene detection using the GCaMP forward (5′-ACGATAAGGATCTCGCCACC-3′) and the GCaMP backward primer (5′-GTCCATGCCGAGAGTGATCC-3′). The β-actin forward (5′-TCCAGCAGATGTGGATCAGCAAGCAGGAG-3′) and the β-actin backward primer (5′-CCGACTGCTGTCACCTTCACCGTTCCAGT-3′) were used for internal control. PCR was performed for 35 cycles of denaturation at 94 °C for 30 s, annealing for 30 s at 59 °C and elongation at 72 °C for 30 s.

To identify transgene integration regions in transgenic marmosets, the Lenti-X integration site analysis kit (Clonetech, Mountain View, CA, USA) was used according to the manufacturer’s protocol. The second round of PCR products were cloned into the pMiniT vector (New England Biolabs, Beverly, MA, USA) for sequencing and alignment of the transgene-flanking sequences was conducted using the BLAST Assembled Genome Database (http://useast.ensembl.org/Callithrix_jacchus/Info/Index and http://blast.ncbi.nlm.nih.gov).

### Reverse transcription-polymerase chain reaction

To determine levels of transgene expression, total RNA was prepared and reverse-transcribed by the High-Capacity cDNA Reverse Transcription kit (Applied Biosystems, Foster City, CA, USA). PCR was performed using GCaMP forward (5′-ACGATAAGGATCTCGCCACC-3′) and GCaMP backward (5′-GTCCATGCCGAGAGTGATCC-3′) primers for GCaMP and β-actin RT forward (5′-AGCAGTCGGTTGGAGCGAGCAT-3′) and β-actin RT backward (5′-TGGCTTTTGGGAGGGCAAGGGA-3′) primers for internal transcript control^74^. PCR was performed for 35 cycles of denaturation at 94 °C for 30 s, annealing for 30 s at 59 °C for GCaMP primers or 62 °C for β-actin primers, and elongation at 72 °C for 30 s.

### GCaMP expression in the transgenic marmosets

Emission of orange fluorescence expression was monitored from the whole body of transgenic marmosets using a FS/ULS-3GN1 head light source (Biochemical Laboratory Services, Budapest, Hungary) by illumination at 525–555 nm and detected by an emission filter with a maximal transmittance wavelength of 557–590 nm. The green fluorescence was produced by illumination at 460–495 nm with a FS/ULS-02B2 head light source (Biochemical Laboratory Services) and detected by an emission filter with a maximal transmittance wavelength of 500–515 nm.

Primary cell cultures for calcium signaling measurements were established from aspirated follicular fluid after oocyte harvesting from TG-Y. Cells were cultured in Dulbecco’s modified Eagle’s medium (DMEM, Invitrogen) containing 10% fetal bovine serum (FBS, Invitrogen), 1% penicillin/streptomycin, and 1mM glutamax (Invitrogen) at 38 °C, in a humidified atmosphere of 5% CO_2_ and 95% air. The media was changed every 2–3 days during the culture period. To test GCaMP functionality in primary cells derived from transgenic marmosets, intracellular calcium signaling was evoked by elevation of the extracellular ATP level through the careful addition of 100mM ATP in DMEM (final concentration of 1 mM). Calcium imaging analyses were performed by detecting the changes in fluorescence intensities continuously at 7 Hz with a confocal laser-scanning microscope LSM 5 Pascal (Carl Zeiss). Cells were excited with 488 nm laser beams and observed through 510–530 emission filter. Images were analyzed with ImageJ software (NIH, Bethesda, MD).

### Flow cytometry of GCaMP transgene expression in blood

ACK lysis solution was added to peripheral blood samples and incubated for 5 minutes, and then cells were diluted and washed with 1X PBS. The pellet was incubated with the alexa488 conjugated rabbit anti-GFP antibody (Invitrogen) and incubated for 30 min on ice. The sample was washed with PBS and resuspended in 200 ul of paraphenylinediamine solution. The cells were analyzed for forward and side scatter, and for green fluorescence (FL-1 channel) using a FACSort (BD Immunocytometry System, San Jose, CA, USA) with CellQuest software.

### Immunohistochemistry

Tissues from transgenic marmosets were fixed in 4% paraformaldehyde (PFA) in PBS overnight at 4 °C. Tissues were embedded in OCT compound, and sliced into 40 μm sections. Alexa488 conjugated rabbit anti-GFP antibody (Invitrogen, 1:1,000) was used to enhance the GCaMP fluorescence and costained with an antibody against the neuronal marker NeuN (EMD Millipore, 1:500). Briefly, sections were incubated with the blocking buffer (5% normal donkey, and 0.2% Triton X-100 in 1X PBS) for 1 hr at room temperature and then incubated with alexa488 conjugated rabbit anti-GFP antibody and mouse anti-NeuN antibody overnight at 4 °C. After incubation with the first antibody, sections were washed with 1X PBS three times for 20 min each, followed by incubation with Alexa 647-conjugated goat anti-mouse secondary antibody (Invitrogen) for 1 hr at room temperature and then washed with 1X PBS. Sections were transferred onto slides, mounted with 0.1% paraphenylinediamine in 90% glycerol/PBS, and imaged with confocal laser-scanning microscope LSM 5 Pascal (Carl Zeiss). AlexaFluor 488 and 647 were excited with 488 and 633 nm laser beams and observed through 510–530 and X650-nm emission filters, respectively.

### Confirmation of germline transmission

After the two female transgenic founder animals reached puberty, serum progesterone concentration was monitored for detecting the onset of their estrus cycles. The estrus cycle was restarted by injecting the cloprostenol (Estrumate) and subjected to superovulation treatment for surgical collection of the oocyte-cumulus complexes (COCs) as described previously^23^. *In vitro* matured oocytes were inseminated with freshly collected wild type sperm (final concentration of 5 × 10^6 ^sperm/ml) for 16–20 h and fertilized embryos were cultured in ISM1 medium (Origio MediCult Meia)^20^,^73^. Transgene integration was confirmed by PCR using the GCaMP forward (5′-ACGATAAGGATCTCGCCACC-3′) and the GCaMP backward primer (5′-GTCCATGCCGAGAGTGATCC-3′). The β-actin forward (5′-TCCAGCAGATGTGGATCAGCAAGCAGGAG-3′) and the β-actin backward primer (5′-CCGACTGCTGTCACCTTCACCGTTCCAGT-3′) were used for internal control. PCR was performed for 37 cycles of denaturation at 94 °C for 30 s, annealing for 30 s at 59 °C and elongation at 72 °C for 30 s. Emission of fluorescence expression was detected using an inverted microscope equipped with a digital camera (AxioCam MRm, Carl Zeiss) and transgene expressing embryos were nonsurgically transferred to synchronized recipient females^23^,^46^,^72^.

## Additional Information

**How to cite this article**: Park, J. E. *et al*. Generation of transgenic marmosets expressing genetically encoded calcium indicators. *Sci. Rep.* **6**, 34931; doi: 10.1038/srep34931 (2016).

## Supplementary Material

Supplementary Information

## Figures and Tables

**Table 1:** Summary of embryo collection procedures.

	Oocytes collected via laparotomy (IVF)	Naturally fertilized embryos (NAT)
Number of procedures	17	52
Procedures with successful collection (%)	17 (100)	43 (82.7)
Total number of embryos collected (Mean number of embryos per procedure)	—	108 (2.5)
Number of GV oocytes (Mean number of oocytes per procedure)	787 (46)	—
Number of matured (MII) oocytes (% maturation rate)	625 (79.4)	—
Number of fertilized oocytes (% fertilization rate)	551 (88.2)	—
Number of embryos subjected to lentiviral injections	476	100
Number of developed embryos(% cleavage rate)	385 (80.9)	—
GCaMP expression confirmed after lentiviral injections (% expression rate)	345 (89.6)	—
Number of embryos used for ET	187	88
Number of surrogates	51	31
Number of pregnancies (% pregnancy rate)	13 (25.5)	9 (29)
Number of deliveries (% delivery rate)	4 (7.8)	3 (9.7)
Number of TGs (% TG per ET)	5 (9.8)	3 (9.7)

ET, embryo transfer; GV: germinal vesicle; MII: metaphase II; TG: transgenic animals; —: not applicable.

**Table 2:** Summary of statistics for production of transgenic marmosets.

Viral construct	Collection method	Embryos transferred	ET procedures	Confirmed pregnancies (%)	Spontaneous miscarriages	Successful deliveries	Live births (% birth rate)	Animal ID
CMV-GCaMP5g	NAT	16	6	3 (50)	1	2	2 (33.3)	TG-Y, TG-D2
	IVF	57	16	5 (10)	5	0	0 (0)	
	Total	73	22	8 (36.4)	6	2	2 (9.1)	
CMV-mKO-GCaMP6s	NAT	22	8	2 (25)	1	1	1 (13)	TG-E
	IVF	6	1	0 (0)	0	0	0 (0)	
	Total	28	9	2 (30)	1	1	1 (11.1)	
hSyn-mKO-GCaMP6s	NAT	30	11	3 (27)	3^a^	0	0 (0)	
	IVF	35	9	3 (33)	1	2	3 (33)	TG-S, TG-L, TG-D1
	Total	65	20	6 (30)	4	2	3 (15)	
hSyn-GCaMP5g	NAT	7	2	0 (0)	0	0	0 (0)	
	IVF	50	14	2 (14)	1	1	1 (7.1)	TG-D3
	Total	57	16	2 (12.5)	1	1	1 (6.3)	
CMV-GCaMP6s	NAT	13	4	1 (25)	1	0	0 (0)	
	IVF	11	4	2 (50)	1	1	1 (25)	TG-J
	Total	24	8	3 (37.5)	2	1	1 (12.5)	
hSyn-GCaMP6s	IVF	28	7	1^b^ (14.29)	0	ND	ND	
Total		275	82	22 (26.8)	14	7	8 (9.8)	

IVF: Oocytes collected via laparotomy; NAT: Naturally fertilized embryos; ET: embryo transfer; TG: transgenic animal; ND: not yet determined.

^a^Stillborn fetuses were spontaneously miscarried after 102d, 135d, and 134d of gestation. They were named M01, M02, and M03, respectively.

^b^Currently ongoing singleton pregnancy confirmed by ultrasound examination.

## References

[b1] Huber D. . Multiple dynamic representations in the motor cortex during sensorimotor learning. Nature 484, 473–478, doi: 10.1038/nature11039 (2012). 22538608 PMC4601999

[b2] Petreanu L. . Activity in motor-sensory projections reveals distributed coding in somatosensation. Nature 489, 299–303, doi: 10.1038/nature11321 (2012). 22922646 PMC3443316

[b3] Broussard G. J., Liang R. & Tian L. Monitoring activity in neural circuits with genetically encoded indicators. Front Mol Neurosci 7, 97, doi: 10.3389/fnmol.2014.00097 (2014). 25538558 PMC4256991

[b4] Akerboom J. . Optimization of a GCaMP calcium indicator for neural activity imaging. The Journal of neuroscience: the official journal of the Society for Neuroscience 32, 13819–13840, doi: 10.1523/JNEUROSCI.2601-12.2012 (2012). 23035093 PMC3482105

[b5] Chen T. W. . Ultrasensitive fluorescent proteins for imaging neuronal activity. Nature 499, 295–300, doi: 10.1038/nature12354 (2013). 23868258 PMC3777791

[b6] Dana H. . Thy1-GCaMP6 transgenic mice for neuronal population imaging *in vivo*. PloS one 9, e108697, doi: 10.1371/journal.pone.0108697 (2014). 25250714 PMC4177405

[b7] Tian L. . Imaging neural activity in worms, flies and mice with improved GCaMP calcium indicators. Nature methods 6, 875–881, doi: 10.1038/nmeth.1398 (2009). 19898485 PMC2858873

[b8] Smith R. L. . Characterization of promoter function and cell-type-specific expression from viral vectors in the nervous system. Journal of virology 74, 11254–11261 (2000). 11070024 10.1128/jvi.74.23.11254-11261.2000 PMC113226

[b9] Wallace D. J. . Single-spike detection *in vitro* and *in vivo* with a genetic Ca^2+^ sensor. Nature methods 5, 797–804, doi: 10.1038/nmeth.1242 (2008). 19160514

[b10] Nathanson J. L., Yanagawa Y., Obata K. & Callaway E. M. Preferential labeling of inhibitory and excitatory cortical neurons by endogenous tropism of adeno-associated virus and lentivirus vectors. Neuroscience 161, 441–450, doi: 10.1016/j.neuroscience.2009.03.032 (2009). 19318117 PMC2728494

[b11] Watakabe A. . Comparative analyses of adeno-associated viral vector serotypes 1, 2, 5, 8 and 9 in marmoset, mouse and macaque cerebral cortex. Neuroscience research, doi: 10.1016/j.neures.2014.09.002 (2014). 25240284

[b12] Zariwala H. A. . A Cre-dependent GCaMP3 reporter mouse for neuronal imaging *in vivo*. The Journal of neuroscience: the official journal of the Society for Neuroscience 32, 3131–3141, doi: 10.1523/JNEUROSCI.4469-11.2012 (2012). 22378886 PMC3315707

[b13] Atkin S. D. . Transgenic mice expressing a cameleon fluorescent Ca^2+^ indicator in astrocytes and Schwann cells allow study of glial cell Ca^2+^ signals *in situ* and *in vivo*. Journal of neuroscience methods 181, 212–226, doi: 10.1016/j.jneumeth.2009.05.006 (2009). 19454294 PMC3142666

[b14] Diez-Garcia J. . Activation of cerebellar parallel fibers monitored in transgenic mice expressing a fluorescent Ca^2+^ indicator protein. The European journal of neuroscience 22, 627–635, doi: 10.1111/j.1460-9568.2005.04250.x (2005). 16101744

[b15] Chen Q. . Imaging neural activity using Thy1-GCaMP transgenic mice. Neuron 76, 297–308, doi: 10.1016/j.neuron.2012.07.011 (2012). 23083733 PMC4059513

[b16] Madisen L. . Transgenic mice for intersectional targeting of neural sensors and effectors with high specificity and performance. Neuron 85, 942–958, doi: 10.1016/j.neuron.2015.02.022 (2015). 25741722 PMC4365051

[b17] Capitanio J. P. & Emborg M. E. Contributions of non-human primates to neuroscience research. Lancet 371, 1126–1135, doi: 10.1016/S0140-6736(08)60489-4 (2008). 18374844

[b18] Sibal L. R. & Samson K. J. Nonhuman primates: a critical role in current disease research. ILAR journal / National Research Council, Institute of Laboratory Animal Resources 42, 74–84 (2001). 10.1093/ilar.42.2.74 11406709

[b19] Chan A. W. Progress and prospects for genetic modification of nonhuman primate models in biomedical research. ILAR journal / National Research Council, Institute of Laboratory Animal Resources 54, 211–223, doi: 10.1093/ilar/ilt035 (2013). PMC3814401 24174443

[b20] Sasaki E. . Generation of transgenic non-human primates with germline transmission. Nature 459, 523–527, doi: 10.1038/nature08090 (2009). 19478777

[b21] Foecking M. K. & Hofstetter H. Powerful and versatile enhancer-promoter unit for mammalian expression vectors. Gene 45, 101–105 (1986). 3023199 10.1016/0378-1119(86)90137-x

[b22] Kugler S., Kilic E. & Bahr M. Human synapsin 1 gene promoter confers highly neuron-specific long-term transgene expression from an adenoviral vector in the adult rat brain depending on the transduced area. Gene therapy 10, 337–347, doi: 10.1038/sj.gt.3301905 (2003). 12595892

[b23] Takahashi T. . Birth of healthy offspring following ICSI in *in vitro*-matured common marmoset (Callithrix jacchus) oocytes. PloS one 9, e95560, doi: 10.1371/journal.pone.0095560 (2014). 24751978 PMC3994092

[b24] Harlow C. R., Gems S., Hodges J. K. & Hearn J. P. The relationship between plasma progesterone and the timing of ovulation and early embryonic development in the marmoset monkey (Callithrix jacchus). Journal of Zoology 201, 273–282, doi: 10.1111/j.1469-7998.1983.tb04276.x (1983).

[b25] Tardif S. D. . Reproduction in captive common marmosets (Callithrix jacchus). Comparative medicine 53, 364–368 (2003). 14524412

[b26] Chaplin T., Yu H., Soares J., Gattass R. & Rosa M. G. P. A conserved pattern of differential expansion of cortical areaas in simian primates. Journal of Neuroscience 18, 15120–15125 (2013). 10.1523/JNEUROSCI.2909-13.2013 PMC6618405 24048842

[b27] Mitchell J. F. & Leopold D. A. The marmoset monkey as a model for visual neuroscience. Neuroscience research 93, 20–46 (2015). 25683292 10.1016/j.neures.2015.01.008 PMC4408257

[b28] Bendor D. A. & Wang X. The neuronal representation of pitch in primate auditory cortex. Nature 436, 1161–1165 (2005). 16121182 10.1038/nature03867 PMC1780171

[b29] Digby L. J. & Barreto C. E. Social organization in a wild population of Callithrix jacchus: Part 1: Group composition and dynamics. Folia primatologica 61, 123–134 (1993). 10.1159/000156739 8206418

[b30] Digby L. J. Social organization in a wild population of Callithrix jacchus. Part 2: Intragroup social behavior. Primates 36, 361–375 (1995).

[b31] Voelkl B. & Huber L. Imitation as faithful copying of a novel technique in marmoset monkeys. PloS one 2(7), e611 (2007). 17622356 10.1371/journal.pone.0000611 PMC1905941

[b32] Huber L. & Voelkl B. In The Smallest Anthropoids: The Marmoset/Callimico Radiation. (eds Ford S. M., Porter L. M. & Davis L. C.) 183–201 (Springer Verlag, 2009).

[b33] Miller C. T. . Marmosets: A Neuroscientific Model of Human Social Behavior. Neuron 90, 219–233, doi: 10.1016/j.neuron.2016.03.018 (2016). 27100195 PMC4840471

[b34] Bourne J. A. & Rosa M. G. Hierarchical development of the primate visual cortex, as revealed by neurofilament immunoreactivity: early maturation of the middle temporal area (MT). Cerebral cortex 16, 405–414, doi: 10.1093/cercor/bhi119 (2006). 15944371

[b35] Hikishima K. . Atlas of the developing brain of the marmoset monkey constructed using magnetic resonance histology. Neuroscience 230, 102–113, doi: 10.1016/j.neuroscience.2012.09.053 (2013). 23047019

[b36] Sawada K. . Fetal sulcation and gyrification in common marmosets (Callithrix jacchus) obtained by *ex vivo* magnetic resonance imaging. Neuroscience 257, 158–174, doi: 10.1016/j.neuroscience.2013.10.067 (2014). 24220690

[b37] Tardif S. D., Mansfield K. G., Ratnam R., Ross C. N. & Ziegler T. E. The marmoset as a model of aging and age-related diseases. ILAR journal / National Research Council, Institute of Laboratory Animal Resources 52, 54–65 (2011). 10.1093/ilar.52.1.54 PMC3775658 21411858

[b38] Kang Y. . CRISPR/Cas9-mediated Dax1 knockout in the monkey recapitulates human AHC-HH. Hum Mol Genet 24, 7255–7264, doi: 10.1093/hmg/ddv425 (2015). 26464492

[b39] Niu Y. . Generation of gene-modified cynomolgus monkey via Cas9/RNA-mediated gene targeting in one-cell embryos. Cell 156, 836–843, doi: 10.1016/j.cell.2014.01.027 (2014). 24486104

[b40] Ke Q. . TALEN-based generation of a cynomolgus monkey disease model for human microcephaly. Cell Res, doi: 10.1038/cr.2016.93 (2016). PMC5034111 27502025

[b41] Sato K. . Generation of a Nonhuman Primate Model of Severe Combined Immunodeficiency Using Highly Efficient Genome Editing. Cell Stem Cell 19, 127–138, doi: 10.1016/j.stem.2016.06.003 (2016). 27374787

[b42] Sadakane O. . Long-Term Two-Photon Calcium Imaging of Neuronal Populations with Subcellular Resolution in Adult Non-human Primates. Cell reports 13, 1989–1999, doi: 10.1016/j.celrep.2015.10.050 (2015). 26655910

[b43] Remy S. . The use of lentiviral vectors to obtain transgenic rats. Methods in molecular biology 597, 109–125, doi: 10.1007/978-1-60327-389-3_8 (2010). 20013229

[b44] Grupen C. G. . Effects of ovarian stimulation, with and without human chorionic gonadotrophin, on oocyte meiotic and developmental competence in the marmoset monkey (Callithrix jacchus). Theriogenology 68, 861–872, doi: 10.1016/j.theriogenology.2007.07.009 (2007). 17714774

[b45] Tkachenko O. Y. . Epidermal growth factor effects on marmoset monkey (Callithrix jacchus) oocyte *in vitro* maturation, IVF and embryo development are altered by gonadotrophin concentration during oocyte maturation. Human reproduction 25, 2047–2058, doi: 10.1093/humrep/deq148 (2010). 20573678

[b46] Ishibashi H. . Efficient embryo transfer in the common marmoset monkey (Callithrix jacchus) with a reduced transfer volume: a non-surgical approach with cryopreserved late-stage embryos. Biology of reproduction 88, 115, doi: 10.1095/biolreprod.113.109165 (2013). 23536374

[b47] Ishibashi H. . Ultrasound-guided non-surgical embryo collection in the common marmoset. Reproductive biology 13, 139–144, doi: 10.1016/j.repbio.2013.02.002 (2013). 23719119

[b48] Takabayashi S., Suzuki Y. & Katoh H. Development of a modified artificial insemination technique combining penile vibration stimulation and the swim-up method in the common marmoset. Theriogenology 83, 1304–1309 e1302, doi: 10.1016/j.theriogenology.2015.01.017 (2015). 25732321

[b49] Tomioka I., Takahashi T., Shimada A., Yoshioka K. & Sasaki E. Birth of common marmoset (Callithrix jacchus) offspring derived from *in vitro*-matured oocytes in chemically defined medium. Theriogenology 78, 1487–1493, doi: 10.1016/j.theriogenology.2012.06.024 (2012). 22925648

[b50] Izpisua Belmonte J. C. . Brains, genes, and primates. Neuron 86, 617–631, doi: 10.1016/j.neuron.2015.03.021 (2015). 25950631 PMC4425847

[b51] Lois C., Hong E. J., Pease S., Brown E. J. & Baltimore D. Germline transmission and tissue-specific expression of transgenes delivered by lentiviral vectors. Science 295, 868–872, doi: 10.1126/science.1067081 (2002). 11786607

[b52] Moran S. . Germline transmission in transgenic Huntington’s disease monkeys. Theriogenology 84, 277–285, doi: 10.1016/j.theriogenology.2015.03.016 (2015). 25917881 PMC4631054

[b53] Putkhao K. . Pathogenic cellular phenotypes are germline transmissible in a transgenic primate model of Huntington’s disease. Stem cells and development 22, 1198–1205, doi: 10.1089/scd.2012.0469 (2013). 23190281 PMC3613972

[b54] Bryda E. C. & Bauer B. A. A restriction enzyme-PCR-based technique to determine transgene insertion sites. Methods in molecular biology 597, 287–299, doi: 10.1007/978-1-60327-389-3_20 (2010). 20013241

[b55] Rosenthal A. & Jones D. S. Genomic walking and sequencing by oligo-cassette mediated polymerase chain reaction. Nucleic acids research 18, 3095–3096 (1990). 2349129 10.1093/nar/18.10.3095 PMC330879

[b56] De Ravin S. S. . Enhancers are major targets for murine leukemia virus vector integration. Journal of virology 88, 4504–4513, doi: 10.1128/JVI.00011-14 (2014). 24501411 PMC3993722

[b57] Beard B. C. . Comparison of HIV-derived lentiviral and MLV-based gammaretroviral vector integration sites in primate repopulating cells. Molecular therapy: the journal of the American Society of Gene Therapy 15, 1356–1365, doi: 10.1038/sj.mt.6300159 (2007). 17440443

[b58] Hematti P. . Distinct genomic integration of MLV and SIV vectors in primate hematopoietic stem and progenitor cells. PLoS Biol 2, e423, doi: 10.1371/journal.pbio.0020423 (2004). 15550989 PMC529319

[b59] Kiem H. P. . Long-term clinical and molecular follow-up of large animals receiving retrovirally transduced stem and progenitor cells: no progression to clonal hematopoiesis or leukemia. Molecular therapy: the journal of the American Society of Gene Therapy 9, 389–395, doi: 10.1016/j.ymthe.2003.12.006 (2004). 15006605

[b60] Yang S. H., Cheng P. H., Sullivan R. T., Thomas J. W. & Chan A. W. Lentiviral integration preferences in transgenic mice. Genesis 46, 711–718, doi: 10.1002/dvg.20435 (2008). 18821598 PMC4381762

[b61] Uchida N., Washington K. N., Lap C. J., Hsieh M. M. & Tisdale J. F. Chicken HS4 insulators have minimal barrier function among progeny of human hematopoietic cells transduced with an HIV1-based lentiviral vector. Molecular therapy: the journal of the American Society of Gene Therapy 19, 133–139, doi: 10.1038/mt.2010.218 (2011). 20940706 PMC3017448

[b62] Zhou S. . A self-inactivating lentiviral vector for SCID-X1 gene therapy that does not activate LMO2 expression in human T cells. Blood 116, 900–908, doi: 10.1182/blood-2009-10-250209 (2010). 20457870 PMC2924228

[b63] Ryu B. Y. . An experimental system for the evaluation of retroviral vector design to diminish the risk for proto-oncogene activation. Blood 111, 1866–1875, doi: 10.1182/blood-2007-04-085506 (2008). 17991809 PMC2234041

[b64] Hu J. . Reduced genotoxicity of avian sarcoma leukosis virus vectors in rhesus long-term repopulating cells compared to standard murine retrovirus vectors. Molecular therapy: the journal of the American Society of Gene Therapy 16, 1617–1623, doi: 10.1038/mt.2008.135 (2008). 18578011 PMC2561952

[b65] Addis R. C. . Optimization of direct fibroblast reprogramming to cardiomyocytes using calcium activity as a functional measure of success. Journal of molecular and cellular cardiology 60, 97–106, doi: 10.1016/j.yjmcc.2013.04.004 (2013). 23591016 PMC3679282

[b66] Szebenyi K. . Generation of a Homozygous Transgenic Rat Strain Stably Expressing a Calcium Sensor Protein for Direct Examination of Calcium Signaling. Scientific reports 5, 12645, doi: 10.1038/srep12645 (2015). 26234466 PMC4522653

[b67] Ji G. . Ca^2+^-sensing transgenic mice: postsynaptic signaling in smooth muscle. The Journal of biological chemistry 279, 21461–21468, doi: 10.1074/jbc.M401084200 (2004). 14990564

[b68] Apati A. . Characterization of calcium signals in human embryonic stem cells and in their differentiated offspring by a stably integrated calcium indicator protein. Cellular signalling 25, 752–759, doi: 10.1016/j.cellsig.2012.12.024 (2013). 23305950

[b69] Tallini Y. N. . Propagated endothelial Ca^2+^ waves and arteriolar dilation *in vivo*: measurements in Cx40BAC GCaMP2 transgenic mice. Circulation research 101, 1300–1309, doi: 10.1161/CIRCRESAHA.107.149484 (2007). 17932328

[b70] Szebenyi K. . Visualization of Calcium Dynamics in Kidney Proximal Tubules. Journal of the American Society of Nephrology: JASN, doi: 10.1681/ASN.2014070705 (2015). PMC4625667 25788535

[b71] Hanawa H. . Comparison of various envelope proteins for their ability to pseudotype lentiviral vectors and transduce primitive hematopoietic cells from human blood. Molecular therapy: the journal of the American Society of Gene Therapy 5, 242–251, doi: 10.1006/mthe.2002.0549 (2002). 11863413

[b72] Thomson J. A., Kalishman J. & Hearn J. P. Nonsurgical uterine stage preimplantation embryo collection from the common marmoset. Journal of medical primatology 23, 333–336 (1994). 7897640 10.1111/j.1600-0684.1994.tb00295.x

[b73] Gilchrist R. B., Nayudu P. L. & Hodges J. K. Maturation, fertilization, and development of marmoset monkey oocytes *in vitro*. Biology of reproduction 56, 238–246 (1997). 9002655 10.1095/biolreprod56.1.238

[b74] Shimamoto Y. . Selection of suitable reference genes for mRNA quantification studies using common marmoset tissues. Molecular biology reports 40, 6747–6755, doi: 10.1007/s11033-013-2791-0 (2013). 24068436

