# Supplementary material for: Generation of transgenic marmosets expressing genetically encoded calcium indicators
Source: Sci Rep. 2016 Oct 11;6:34931. doi: 10.1038/srep34931 (PMC5057151; doi:10.1038/srep34931)

Supplementary Information

Generation of transgenic marmosets expressing genetically encoded calcium indicators

Jung Eun Park[1], Xian Feng Zhang[1], Sang-Ho Choi[1], Junko Okahara[2], Erika Sasaki[2, 3], Afonso C. Silva[1,*]

[1] Cerebral Microcirculation Section, Laboratory of Functional and Molecular Imaging, National Institute of Neurological Disorders and Stroke, National Institutes of Health, Bethesda, MD, USA

[2] Department of Applied Developmental Biology, Central Institute for Experimental Animals, Tonomachi, Kawasaki, Kanagawa 210-0821, Japan

[3] Keio advanced Research Center, Keio University, Shinanomachi, Shinjuku-ku, Tokyo 160-8582, Japan

Supplementary Table S1. Genomic integration loci of the transgene in transgenic marmosets.

Tg ID	Genomic location	Overlapping genes	Length	%ID	Sequence (Bold: marmoset sequence, Italic: lentiviral sequence)
TG-Y	9:64243780	ATXN7L3B	511	99.61	**CCCTCTCCGCAGTCGTTTACAAATTTAAA**GCTTGGAAA*TATTTTTTTGCATCAGTTTCGT*
TG-E	2:197330287	Intergenic region	218	100	*ATCTTGTCTTCGTTGGGAGTGAATTAGCCCTTCC***ACTAACCGAGTACCAGAAATTGGTTAATACACCCTTGTTAGTTGAATGAACAATGGCCCATTTCAAAAAGAAAGAAGCCAGTAATCTTGTGGGAGGA**
	7:136607274	RTCA	159	100	*GCAAAAAGCAGATCTTGTCTTCGTTGGGAGTGAATTAGCCCTTCCA***ATTATCCAGTGGTTTTAACATCTATTGATGATTTTTCTCTGCATCAGTTATTTCATT**
	11:32419859	NCAPD3	540	99.81	*GAGAGACCCAGTACAAGCAAAAAGCAGATCTTGTCTTCGTTGGGAGTGAATTAGCCCTTCC*G**ATCCTATAATGCACAGGAAAAGATTTCTTAAAGATACTTTAATCATTAAGAAAACCACTGTCAAAGTGAATTTATCAAAA**
	16:72233830	Intergenic region	142	89.44	*ATATAAGCAGCGCGTTTTGCCTGTACTGGGTCTCTCTGGTTAGACCAGATCTGAGCCTGGGAGCTC*GAATTCTCTGGCCGTCG**TTTTACAACGTCGTGACTGGGAAAACCCTGGCGTTACCAACTTAATCGCCTTGC**
TG-S	2:197330288	Intergenic region	206	100	**GTGTATTAACCAATTTCTGGTACTCGGTTAG***TGGAAGGGCTAATTCACTCCCAACGAAGACAAGATCTGCTTTTT*
	9:35209886	BICD1	435	99.77	**ACAATGAACTAAATGAATACTGACAGCAGGGTTTCTGATTTACTTATCTCATTAT***TGGAAGGGCTAATTCACTCCCAACGAAGACAA*
	11:107855778	Intergenic region	720	99.58	*AGCAGATCTTGTCTTCGTTGGGAGTGAATTAGCCCTTCCA***TACACAAATAAAGCAAATTATCCAGGCATGGTGGCACGTGCTTGAAGTCCCAGCT**
TG-L	1:126737526	Intergenic region	820	98.66	*AAAAGCAGATCTTGTCTTCGTTGGGAGTGAATTAGCCCTTCC***AGAACTCATCCTTTTTTATGGCTCCATAGTATTCTATGGTGTATGTGTGCCATATTTTCTTTATCTAGTCTATCATTGATGGGCATTTGGGTTGGCT**
	2:72750672	MA_M002_JSM116D85R	460	99.78	**AGGTTGCAGTGAGCCAAGATAGTGTCACTGAACTCCATCCTGGGCAACACAGCAAGACTGTGTCTCAAAAAAACAAAATAGGCTG***TGGAAGGACTAATTCACTCCCAACGAAGACAAGATCTGCTTTTTGCTTGTACTGGGTCTCTCTGGTT*
	14:80829389	ZNF512	528	99.62	*TACAAGCAAAAAGCAGATCTTGTCTTCGTTGGGAGTGAATTAGCCCTTCCA***AAACCCCATTAAACAAAGGAAAGGGGATAAAAATAAATTTTTTT**
	15:33065540	IL17RD	208	99.04	*GATCTGGTCTAACCAGAGAGACCCAGTACAAGCAAAAAGCAGATC*TTGTCTTCGTTGGGAGTGAATTAGCCCTTCCA**GGTTTCTGGTGTCTTTGATGACACACGATGTCTCGGAACAAAA**
TG-D1	6:47458599	TLK1	510	99.22	*CTTGTCTTCGTTGGGGGTGAATTAGCCCTTCCA***GATAGGAAACCCCAATTAAGTGAGGTACTATCTTCTCAAAGAATTTCATTCTTTTCA**
	7:67759963	Intergenic region	523	99.81	**GAATTCACCTCTATATAATTCATCTATGTAACAAAAACCACTCGTACC***TGGAAGGGCTAATTCACTCCCAACGAAGACGAGAT*
TG-D2	2:110737191	LNPEP	705	100	*CGGGCACACACTACTTGAA*G**AACCTAAAACATTCAGGAAAGTGAACAGTCCCAACTTACAAGTGACTATGAATTTTCTGACTATGAAAGAGA**
	6:47458599	TLK1	76	100	*ATCTTGTCTTCGTTGGGAGTGAATTAGCCCTTCCA***GATAGGAAACCCCAATTAAGTGAGGTACTATCTTCTCAAAGAATTTCATTCTTTTCATTAGCAGACTTCTGTTTTA**
	7:100483224	JAK1	155	100	*TCTCTGGTTTCCCTTTCGCTTTCAAGTCCCTGTTCGGGCGCCACTGCTAGAGATT***TTCAAAGTTCACGAGCCCCAGGCACATGCTCAGCCTTCCTCCAAGTTTAATCATAAACAGGCAAGATAAACAGATGTCCAAGGAAA**
TG-J	14:80829389	ZNF512	46	100	*AAGCAAAAAGCAGATCTTGTCTTCGTTGGGAGTGAATTAGCCCTTCCA***AAACCCCATTAAACAAAGGGAAAGGGGATAAAAATAAATTTTTTTA**
	20: 43696763	ZNF778	295	99.32	**TACCTGTGTGCGCCACCACACCTGCCTAACTTTGTACTCTTAGTAGAGACGGGGTTTC***TGGAAGGGCTAATTCACTCCCAACGAAGACAA*
TG-D3	6:124325749	PIKFYVE	683	100	*CCAGTACAAGCAAAAAGCAGATCTTGTCTTCGTTGGGAGTGAATTAGCCCTTC***CACTATGAAAGTAAATTTTCATGCATTACTTCCTTTTATACCCATAATAGCCACATAATAATAATTAGACATCCTTATTTTTCAGAAAAT**
	15:40004695	THOC7	155	100	*AAAAGCAGATCTTGTCTTCGTTGGGAGTGAATTAGCCCTTCCA***TCAGGCAACCACTATATCATTCTAATCCATTTTTA**

Supplementary Figure S1. Transgene expression in blood. Results of FACS analysis using whole peripheral blood cells and leukocyte subsets from transgenic marmosets. The percentage of GCaMP-positive cells is shown in the top right of each panel.

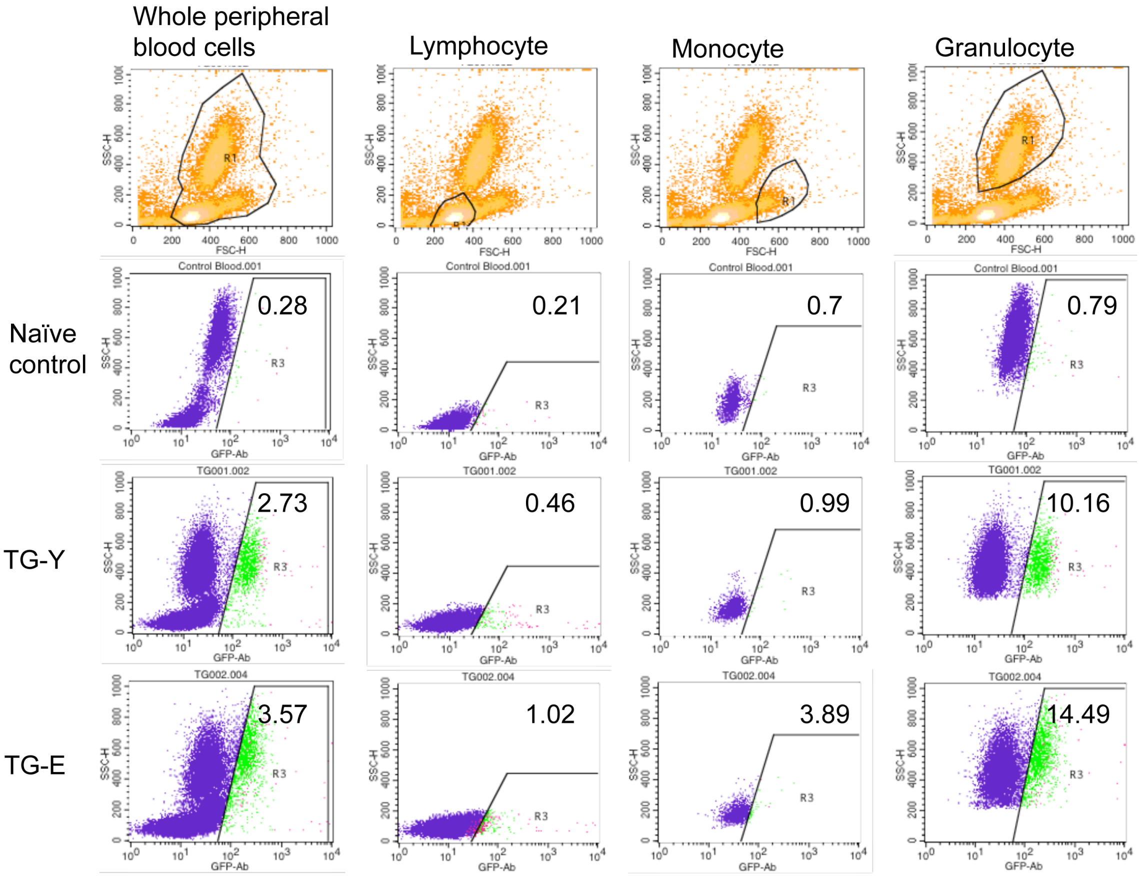

Supplementary Figure S2. Stimulus evoked response of fibroblast cells derived from CMV-mKO-GCaMP6s (TG-E) and Syn-mKO-GCaMP6s (TG-D1) transgenic marmosets. (A-B) Images of GCaMP labeled cells derived from TG-E skin tissue before (A) and after (B) stimulation with calcium chloride show robust changes in fluorescence after the increase in intracellular calcium levels. (a-e) Time course of the changes in fluorescence in five example areas demarcated in A. (C-D) Images of GCaMP labeled cells derived from TG-D1 spinal cord tissue before (C) and after stimulations with calcium chloride. (f-j) Time course of the changes in fluorescence in five example areas demarcated in in C.

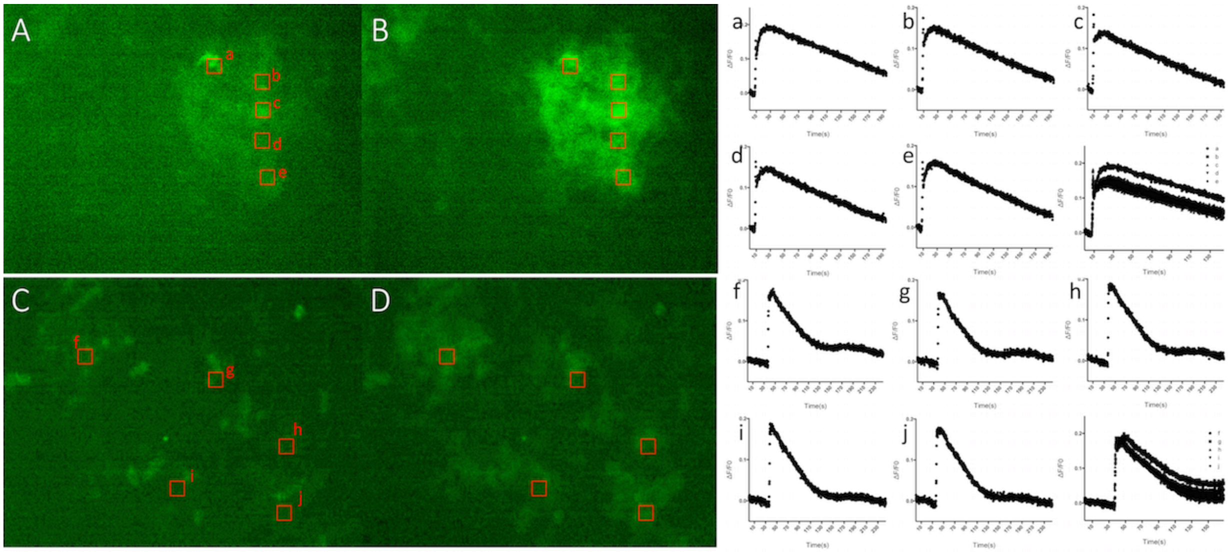

Supplement: Supplementary Information [file srep34931-s1.pdf]
